# Maintaining consistent bladder filling during external beam radiotherapy for prostate cancer

**DOI:** 10.1016/j.tipsro.2021.01.002

**Published:** 2021-01-30

**Authors:** Nicola J. Nasser, Eyal Fenig, Jonathan Klein, Abed Agbarya

**Affiliations:** a Department of Radiation Oncology, University of Maryland School of Medicine, Maryland Proton Treatment Center, Baltimore, MD, USA; b Institute of Oncology, Davidoff Center, Rabin Medical Center, Beilinson Hospital, Petah Tikva, Israel; c Department of Radiation Oncology, Montefiore Medical Center and Albert Einstein College of Medicine, Bronx, NY, USA; d Institute of Oncology, Bnai Zion Medical Center, Haifa, Israel

**Keywords:** Radiation therapy, Bladder filling, Prostate cancer, Urinary catheter, Check valve, Float

## Abstract

Radiation therapy for patients with prostate cancer is preferably provided with a full urinary bladder. Full bladder can potentially move the small intestine out of the radiation treatment regions, and results in decreased small bowel radiation dose and gastrointestinal toxicity. Maintaining consistent bladder filling during computerized tomography simulation scan used for treatment planning and at daily radiation treatments is challenging. Here we present an in-development urinary catheter with a floating balloon that drains the bladder only when urine reaches to a prespecified level, and review current methods used in clinic to ensure consistent bladder filling. These includes bladder filling protocols, ultrasound scanning and biofeedback techniques.

## Highlights

• Radiation for prostate cancer is preferably provided with a full urinary bladder.
• There are discrepancies how well current methods achieve consistent bladder filling.
• A urinary catheter with a check-valve controlled by a float is under development.

## Graphical abstract

**Figure:**
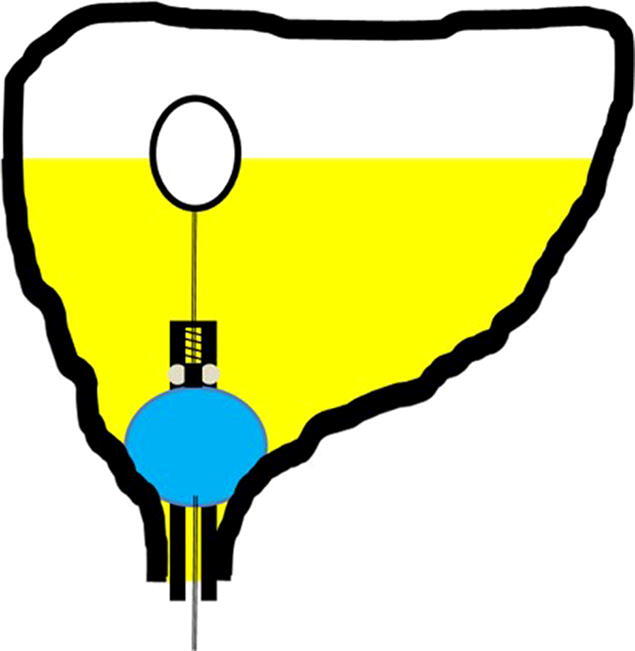

## Introduction

Prostate cancer is the most common non-cutaneous malignancy among men [1]. Curative treatment of non-metastatic prostate cancer is surgical resection and/or radiation therapy (RT), with or without hormonal therapy [2]. External beam RT (EBRT) for prostate cancer is preferentially delivered with a full bladder [3]. When the urinary bladder fills, it can push part of the small intestine lying just above it superiorly and potentially out of the radiation therapy fields. The location of the prostate could be substantially affected by the extent of the urinary bladder and rectum fillings [3], [4], [5], [6].

Standard fractionation EBRT for treatment of prostate cancer is delivered using daily radiation doses of 1.8–2 Gy, five days a week, over 8–10 weeks, up to a total dose of 86.4 Gy [7]. The radiobiologic characteristics of prostate cancer, which has low alpha/beta ratio, provided the theoretical basis for hypofractionated RT [8]. The CHHiP trial found that a moderately hypofractionated protocol providing 60 Gy in 3 Gy daily doses, five days a week was non inferior to conventional fractionation using 74 Gy in 37 fractions [9]. Stereotactic Body Radiation Therapy (SBRT) providing radiation over five fractions is non-inferior to standard fractionation EBRT [10], [11], [12], [13]. 2STAR phase 2 trial found that SBRT delivering 26 Gy in two fractions for localized prostate cancer is safe and feasible [27], and another study reported that providing 24 Gy to the prostate in a single fraction is safe [14].

Delivering radiation therapy to the prostate over a limited number of fractions, with high doses per fraction, necessitates a highly accurate image guided radiation therapy, to ensure that the radiation is delivered to the prostate while sparing surrounding organs. Moreover, the continuous accumulation of urine in the bladder from the initial patient setup and cone beam imaging until the end of radiation delivery, could result in intrafraction error, with potentially lower doses to the clinical target volumes and higher doses to the organs at risk, as a result from the change in bladder filling. Variations in bladder filling have been shown to affect target coverage is several studies [3], [4], [15], [16], [17], [18], [19].

Here, we review methods to maintain consistent amount of urine in the bladder at the time of CT simulation scan for RT planning, and at each time RT is delivered, and describe an in-development urinary catheter with a check valve controlled by a floating balloon that drains the bladder only when urine reaches a prespecified level.

### Bladder filling protocols

Bladder filling protocols specify a policy of urine voiding and water drinking before RT, with the aim of achieving consistent urine volume in the bladder each day at time of RT. There is no consensus on what bladder filling protocols should be used for prostate external beam radiotherapy.

Braide et al. [6] compared two bladder filling protocols in patients receiving salvage RT after radical prostatectomy and prostate specific antigen (PSA) relapse. Patients were instructed to void their bladder of urine and then drink 300 ml of water one hour before radiation (group 1) or maintain a comfortably filled bladder (Group 2). The bladder volumes were calculated based on the planning CT and a weekly Cone Beam CT (CBCT). Neither bladder filling protocols managed to achieve consistent bladder fillings for RT [6].

### Ultrasound scanning and biofeedback techniques

Ultrasound scanning of the bladder is a validated method to evaluate the bladder volume. O'Shea et al. found that bladder volume measurements obtained via ultrasound were not significantly different from the volumes delineated on the planning CT scan, with a mean difference of 9.65 ml, p = 0.351 [20]. Cramp et al. [21] reported a protocol using repeated bladder scanning in intervals of 15 min before RT, aiming to achieve a bladder urine volume of 250 ml. Ninety three percent of patients in the bladder scan group were ready for treatment after the CBCT, compared to 75% of the patients who were not on the bladder scan measurement protocol (p < 0.0001) [21]. Thus, this method resulted in less treatment delays after CBCT and less need for reimaging before treatment delivery.

Hynds et al. [22] assessed the daily consistency of bladder filling using ultrasound scanner in men receiving radical three-dimensional conformal radiotherapy for prostate cancer. The patients were instructed to void the bladder and then drink 500 ml of water within the next 15 min, and thirty minutes later to proceed with radiotherapy [22]. The bladder-filling protocol failed to provide reproducible and consistent bladder volumes from the time of planning through the daily treatments, with the urine volume at CT planning larger than the volume achieved during daily RT treatments [22].

Stam et al. [5] tested the use of bladder ultrasound and biofeedback for optimizing bladder filling. The feedback consisted of telling the patient his daily bladder volume together with a drinking advice. When patients had a bladder urine volume ranging from 80% to 120% of the intended volume, they were instructed to drink the same amount of water the next day. The bladder filling and daily variations did not significantly differ between the control and the feedback group [5]. Gawthrop et al. [23] found a good correlation between bladder filling as measured on CBCT, and the bladder scan, with a Pearson’s correlation coefficient of 0.85. Patients who reported “comfortably full” bladder at time of CBCT were found to have adequately full bladder in 76.5% of the times only [23], indicating the need for bladder filling validation before treatment.

### Empty bladder as a strategy for reproducing consistent bladder filling

Due to the challenges of obtaining consistently full bladder, some groups proposed utilizing consistently empty bladder when treating the prostate gland and seminal vesicles only with RT [14], [24], [25]. Chetiyawardana et al. reported that empty bladder filling protocol of EBRT for localized prostate cancer resulted in non-inferior treatment outcomes compared to patients treated with full bladder [24]. Greco et al. utilized a Foley catheter to empty the bladder during 24 Gy single fraction RT to the prostate, and reported that the treatment can be safely delivered, with low acute toxicity [14].

### Nasser - Zelefsky catheter

**Fig. 1:**
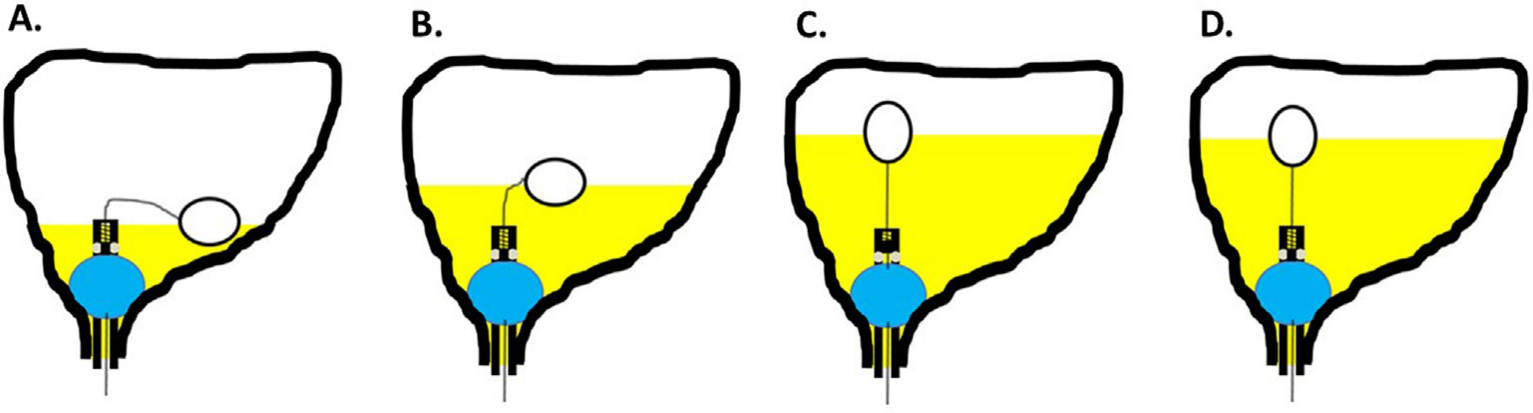
Schematic drawing of the catheter described by Nasser and Zelefsky. The urinary catheter drains the bladder only when urine reaches a predefined level. First balloon fills with water (blue); second balloon fills with air and function as a float (white). A. bladder is almost empty, valve close; B. bladder is partly full, valve close; C. bladder full, valve open; D. catheter drained part of the urine, valve close. (For interpretation of the references to color in this figure legend, the reader is referred to the web version of this article.)

**Fig. 2:**
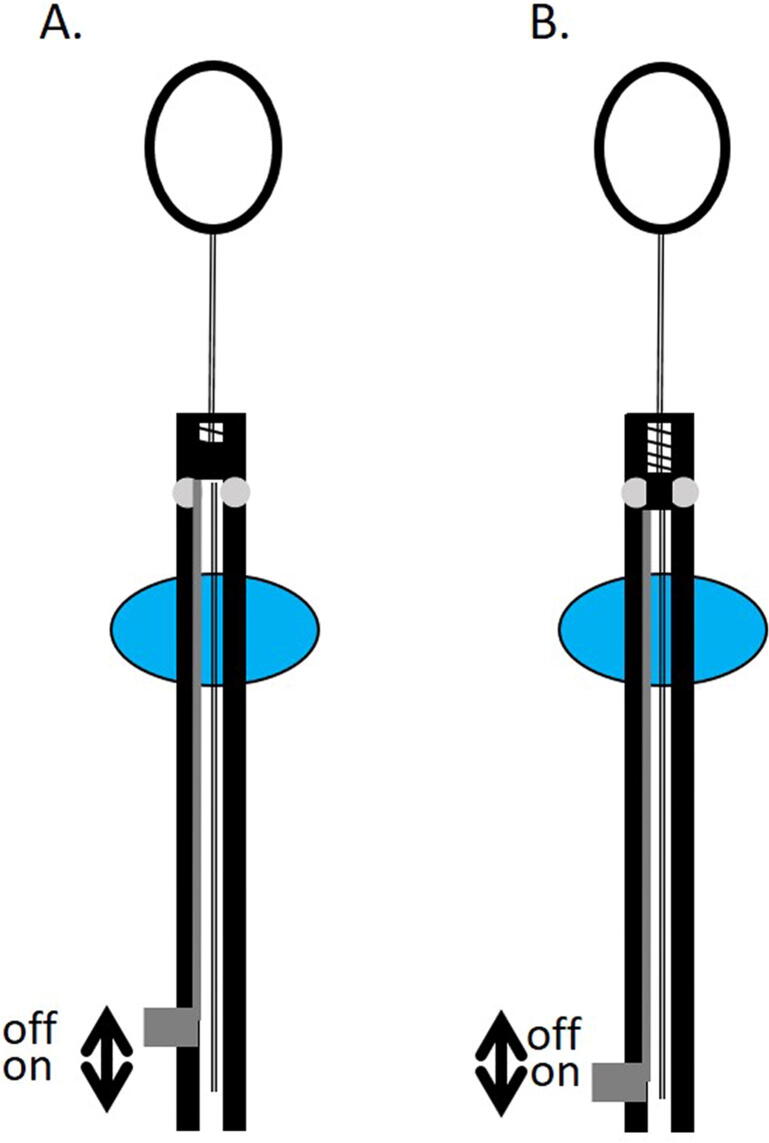
Schematic drawing of the Nasser-Zelefsky catheter with a deactivation mechanism. A. The filling mechanism is deactivated by continuously compressing the spring in the check-valve allowing continuous urine drainage. B. The filling mechanism is activated, and the spring in the check-valve is fully controlled by the floating balloon as a function of urine level.

A catheter with a check valve controlled by a float, which aims to keep the urinary bladder full to a specific urine level, and drains the excess urine produced, has been described by Nasser and Zelefsky and is currently in preclinical development phase [26]. This device is a catheter that has two balloons (Fig. 1). The catheter is designed to be inserted by a medical provider, after which a first balloon is filled by the provider with water and anchors the catheter to the bladder, and the second balloon is filled with air, allowing it to float on the urine. The floating balloon is attached to a spring-loaded check-valve and drains the bladder by opening the valve only when urine reaches a specific predefined level. The predefined level of bladder filling is determined by the length of the string connecting the floating balloon to the valve (Fig. 1). The catheter has a deactivation mechanism that allows the bladder to be empty during the day when the patient is out of hospital, by continuously compressing the spring in the check valve (Fig. 2). The filling mechanism is designed to be activated 2–3 h before the treatment, by the patient. The catheter was tested in phantom models only, and further validation is needed in animal models and clinical trials. The main drawback of this technique is the need for an indwelling catheter. The Memorial Sloan Kettering Cancer Center filed a patent application, an international search report was conducted, and was published by the World International Property Organization, that did not found a similar prior art [26].

## Discussion

Maintaining consistent urinary bladder filling during radiation therapy for prostate cancer is important for accurate treatment delivery. The current protocols for bladder filling provide limited reproducibility of the target urine volume and do not account for changes in urine volume during pretreatment imaging and radiation delivery. Nasser-Zelefsky catheter (Fig. 1) is a unique novel invention currently in preclinical development, that could potentially provide accurate, consistent, and reproducible bladder filling during radiation therapy for prostate cancer, with a main drawback of the need to insert a catheter to the patient. The challenges of developing this catheter is the high cost of production of clinically suitable prototypes, and the cost of preclinical and clinical trials needed for licensure.

RT for prostate cancer is preferably delivered with the urinary bladder full. This helps keep the bowel out of the high dose radiation regions and decreases gastrointestinal toxicity. The challenge of having the patients on the treatment table with the bladder consistently full for each treatment visit and with bladder filling the same as during the CT planning scan, has been investigated in many studies which suggest that bladder filling is not consistent [4], [5], [15], [16], [19]. Even with established drinking protocols for bladder filling, the volume of urine remained inconsistent between treatment visits [6], [19]. The rate of urine production could be affected by the hydration status of the patient before radiation, as well as other factors such as background diseases (diabetes, renal failure, etc.) and use of medications such as diuretics. Bladder ultrasound scanning before the treatment may need multiple examinations to ensure a prespecified urine threshold is reached [5], and after that, as the patient wait for his turn to get on the treatment table, additional urine could accumulate in the bladder, ending in having some of the patients with difficulties holding their urine. Moreover, the urine bladder scanner is not fully consistent and in many cases is operator dependent.

The inconsistent bladder filling regardless of the protocol used, led multiple groups to investigate treating prostate cancer with radiation therapy on empty bladder. The rationale is that it is easier to obtain consistently empty bladder than a full one, while trying to reduce bowel dose by rigorous treatment planning [14], [24], [25]. Consistent empty bladder throughout the treatment needs continuous drainage with a Foley catheter especially when using ultrahypofrationaed doses [14]. While treating prostate only with empty bladder could be feasible, treating the prostate and pelvic lymph nodes should be preferably done with a full bladder, to limit the radiation dose to the small bowel. Maintaining full bladder during RT could become more challenging toward the end of the RT course due to genitourinary toxicity, especially in patients after radical prostatectomy.

Bladder filling protocols, ultrasound scanning, and biofeedback techniques fall short of achieving consistent bladder filling. CBCT before RT could be necessary for validation of bladder filling, especially before hypofractionated RT is delivered.

## Conclusions

Current bladder filling techniques for patients treated with EBRT result in non-consistent volumes of urine in the urinary bladder at time of treatment. CBCT prior each fraction of RT is useful for urine volume estimation in order to ensure consistent bladder filling for RT. Nasser - Zelefsky catheter, which utilizes a check valve controlled by a float, needs validation in preclinical and clinical studies to test its feasibility and consistency of bladder filling. Further research is needed to develop noninvasive methods of real-time urine measurement that ensure highly consistent bladder filling.

## Declaration of Competing Interest

The authors declare the following financial interests/personal relationships which may be considered as potential competing interests: N.J.N. declare being an inventor on a patent application filed by the Memorial Sloan-Kettering Cancer Center (MSKCC) about the catheter described in the manuscript, US provisional application number 62094123, International Application Number PCT/US2015/066845, International Publication Number WO2016/100901 A1. N.J.N. have a license agreement with MSKCC regarding this invention. E.F., J.K., and A.A. declare that they have no known competing financial interests or personal relationships that could have appeared to influence the work reported in this paper.
